# Expression of novel nitrate reductase genes in the harmful alga, *Chattonella subsalsa*

**DOI:** 10.1038/s41598-018-31735-5

**Published:** 2018-09-07

**Authors:** Yanfei Wang, Josée N. Bouchard, Kathryn J. Coyne

**Affiliations:** 1 University of Delaware, 1044 College Drive, Lewes, DE 19958 USA; 2 Algenol Biotech, 16121 Lee Rd., Suite 100, Ft. Myers, FL 33912 USA

## Abstract

Eukaryotic nitrate reductase (NR) catalyzes the first step in nitrate assimilation and is regulated transcriptionally in response to external cues and intracellular metabolic status. NRs are also regulated post-translationally in plants by phosphorylation and binding of 14-3-3 proteins at conserved serine residues. 14-3-3 binding motifs have not previously been identified in algal NRs. A novel NR (NR2-2/2HbN) with a 2/2 hemoglobin domain was recently described in the alga *Chattonella subsalsa*. Here, a second NR (NR3) in *C*. *subsalsa* is described with a 14-3-3 binding motif but lacking the Heme-Fe domain found in other NRs. Transcriptional regulation of both NRs was examined in *C*. *subsalsa*, revealing differential gene expression over a diel light cycle, but not under constant light. *NR2* transcripts increased with a decrease in temperature, while *NR3* remained unchanged. *NR2* and *NR3* transcript levels were not inhibited by growth on ammonium, suggesting constitutive expression of these genes. Results indicate that *Chattonella* responds to environmental conditions and intracellular metabolic status by differentially regulating *NR* transcription, with potential for post-translational regulation of NR3. A survey of algal NRs also revealed the presence of 14-3-3 binding motifs in other algal species, indicating the need for future research on regulation of algal NRs.

## Introduction

Nitrate and ammonium are the most abundant nitrogen sources that phytoplankton utilize for their growth and metabolism: approximately 40% of global primary production is the result of assimilating these two nitrogenous nutrients by phytoplankton^1^. Even so, nitrogen is a limiting nutrient for phytoplankton in many marine environments and its acquisition and assimilation is highly regulated. In order to utilize nitrate as a nitrogen source, phytoplankton reduce nitrate to ammonium by two sequential reactions that are catalyzed by nitrate reductase (NR), with electrons donated by NAD(P)H generated by the light reactions of photosynthesis, and nitrite reductase, with ferredoxin as an electron donor^2,3^. NR catalyzes the first enzymatic and also the rate-limiting step in nitrate assimilation, and plays a key role in coordinating nitrogen and carbon assimilation^3,4^.

NR gene expression and activity are regulated in response to environmental conditions and intracellular metabolic status^5–9^. The tight regulation of *NR* not only plays an important role for organisms to optimize nitrogen assimilation and adjust different metabolic pathways, but also prevents the accumulation of potentially toxic nitrite and formation of reactive oxygen and nitrogen species^10,11^. In general, both NR gene expression and activity respond to nitrogen source, temperature and light. Changes in *NR* transcript abundance typically reflect changes in NR protein abundance and activity^12,13^. In some species, *NR* gene expression is induced by the presence of nitrate, and repressed or inhibited completely by the presence of ammonium^7^. *NR* gene expression can also be constitutive, as in the raphidophyte, *Heterosigma akashiwo*^14^. Regulation of algal *NR* transcript abundance by temperature has been examined in diatoms, where increased transcription and NR activity in this group of algae may serve as a means to dissipate excess energy under high light and low temperature conditions^4^. *NR* gene expression also varies over a diel cycle^15^. These variations can be driven by an endogenous circadian rhythm, or in response to light- dark cycles, where *NR* gene expression is regulated by metabolic feedback controls^15,16^. For instance, Vergara *et al*. demonstrated that *NR* gene expression in the marine diatom *Thalassiosira weissflogii* was regulated by the photosynthetic organic carbon accumulated in the light period, indicating coordination with photosynthetic activity and light- dark cycle regulation instead of endogenous control by a circadian clock^17^.

NR enzyme activity is also regulated in higher plants at the post-translational level by reversible phosphorylation of a conserved serine (Ser) residue in the hinge 1 region, permitting binding of 14-3-3 proteins^18,19^. Binding of 14-3-3 proteins inhibits NR activity via obstruction of intra-molecular electron flow (i.e. the movement of electrons between domains)^20,21^. According to the published studies to date, the phospho-Ser residue required for the binding of 14-3-3 proteins in NRs of higher plants is absent from NRs of mosses and algae, suggesting alternative mechanisms for regulation of NRs in these species^6,22^. However, the lack of 14-3-3 binding motifs in NRs of algae were only examined in a few species from limited phylogenetic groups, including several green algal species (*Chlamydomonas reinhardtii*^23^, *Volvox carteri*^23,24^, *Chlorella vulgaris*^23,25^, and *Dunaliella tertiolecta*^26^), and diatoms^27^ (*Phaeodactylum triconutum* and *Thalassiosira pseudonana*).

**Figure 1:**
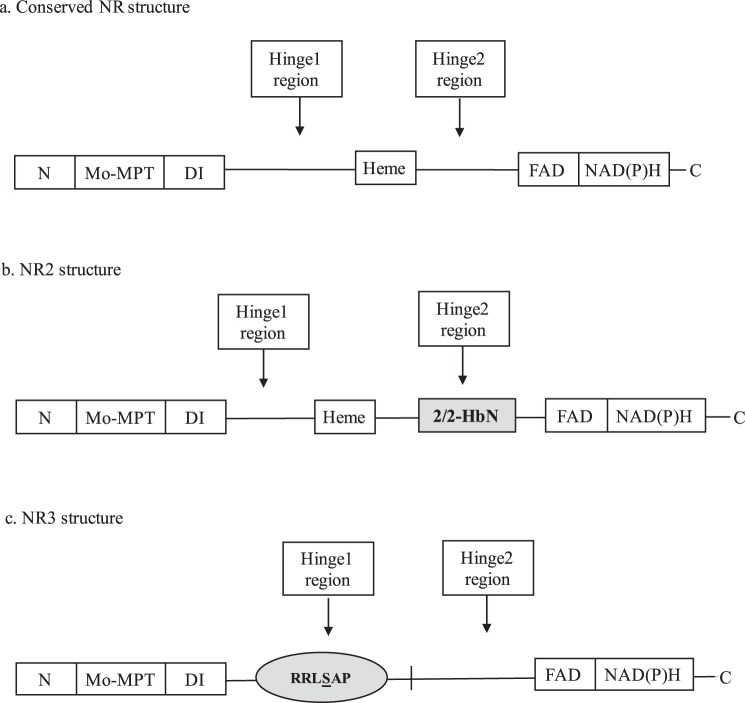
Alternative domain structures for nitrate reductase enzymes. (**a**) The conserved structure of nitrate reductase in higher plants, algae, and fungi, depicting five domains (MO-MPT, DI, Heme-Fe, FAD, and NADH domains) and 3 regions (N-terminal, hinge 1 and hinge 2 regions)^3^. (**b**) The structure of NR2 with a 2/2HbN domain (indicated by grey box) inserted in its Hinge 2 region of *Heterosigma akashiwo* and *Chattonella subsalsa*^2^. (**c**) The structure of NR3 lacking the Heme-Fe domain and with a 14-3-3 protein binding motif (indicated by grey circle) at a phospho-Ser residue (underlined in RRLSAP) in the Hinge 1 region of *Chattonella subsalsa*. N: N-terminal region; Mo-MPT: molybdenum-molybdopterin cofactor domain; DI: dimer interface; Heme: cytochrome b5-binding domain containing Heme-Fe; FAD: flavin adenine dinucleotide domain; NAD(P)H: NAD(P)H domain; C: C-terminal of NR enzyme.

Despite differences in regulation of NRs among species, eukaryotic NRs share a conserved structure constituted by five domains^3^ (Fig. 1a): (a) molybdenum-molybdopterin cofactor domain (Mo-MPT); (b) dimer interface (DI); (c) cytochrome b5-binding domain containing Heme-Fe (Heme-Fe domain); (d) flavin adenine dinucleotide (FAD) domain; and (e) NAD(P)H domain. NR also includes three regions that function to regulate and stabilize the enzyme^3^: the N-terminal region, a hinge 1 region that is located between the DI and Heme-Fe domains, and a hinge 2 region between the Heme-Fe and FAD domains. Recently, a novel nitrate reductase sequence, NR2-2/2HbN (hereafter referred to as NR2), was identified in the harmful algal bloom species *Heterosigma akashiwo* and *Chattonella subsalsa* (Raphidophyceae)^2^ (Fig. 1b). NR2 has a 2/2 hemoglobin (2/2HbN) domain inserted in the hinge 2 region that was not present in the deduced amino acid sequence of *H*. *akashiwo* NR1 (*Hs*NR1)^14^ or in any other eukaryotic NR to date^12,26–28^. Consistent with reports of NR sequences from other algae, a 14-3-3 binding site motif was not identified in the translated amino acid sequences of *Hs*NR1 or the NR2 sequences for either *H*. *akashiwo* or *C*. *subsalsa*^2^.

A second novel NR gene, designated here as NR3, was revealed in the transcriptome of *C*. *subsalsa* as part of the Marine Microbial Eukaryote Transcriptome Sequencing Project^29^ (MMETSP) (Sample Names: MMETSP0947–0950; PI: Kathryn Coyne; https://www.imicrobe.us/#/combined_assemblies/21; accessed on 3/2/2018; sequence ID: CAMPEP_0187150166 in combined assembly peptides). A sequence orthologous to *Hs*NR1 was not identified in the transcriptome of *C*. *subsalsa*. The deduced amino acid sequence of *C*. *subsalsa* NR3 (Fig. 1c) lacks the 2/2HbN domain found in *Cs*NR2 as well as the conserved Heme-Fe domain present in all other eukaryotic NRs. In contrast to *Cs*NR2 and other algal NRs described to date, the deduced amino acid sequence of *NR3* also includes a proline- and serine-rich region in the hinge 1 region, similar to 14-3-3 protein binding domains in plant NRs. The regulation of NR2 and NR3 by environmental conditions and their relative impact on the competitive success of *C*. *subsalsa* in natural phytoplankton communities remain unknown.

The objectives of this investigation were to examine the hypothesis that *NR2* and *NR3* transcript abundances in *C*. *subsalsa* are differentially regulated in response to nitrogen source, temperature and diel changes in light. Mechanisms by which NR3 may retain function without the Heme-Fe domain are also discussed. Finally, a comprehensive survey was conducted to identify 14-3-3 binding motifs in other algal NRs.

## Results

### Sequence analysis of NR3 in *C*. *subsalsa*

**Figure 2:**
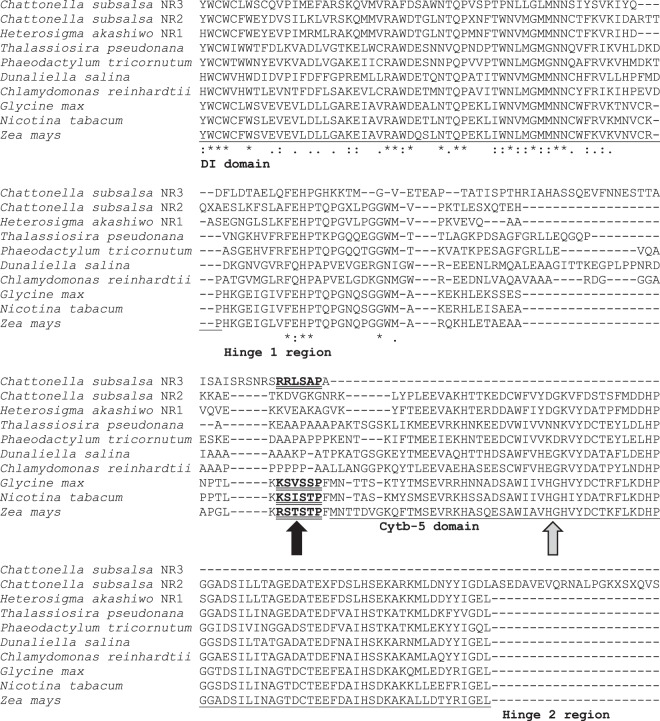
Alignment of the hinge 1 region and adjacent regions of NR3 in *C*. *subsalsa* with NR2 in *C*. *subsalsa*, NR1 of *Heterosigma akashiwo*, NRs of four other representative algal species, *Thalassiosira pseudonana*, *Phaeodactylum tricornutum*, *Dunaliella salina*, and *Chlamydomonas reinhardtii*, as well as NRs in higher plants, *Glycine max*, *Nicotina tabacum*, and *Zea mays*. The single underlined sequences indicate the adjacent domains, showing the absence of the cytb-5 binding domain in NR3 (indicated by grey arrow). The double underlined sequences indicate the motif containing the serine phosphorylation site for 14-3-3 protein binding in NR3 and NRs in higher plants (indicated by black arrow). The identical residues in all sequences are marked by *; the highly and weakly conserved residues are marked by : and ., respectively.

The deduced amino acid sequences of *NR3* cDNA clones in plasmids CsNR3-P2 and CsNR3-P10 were identical to the *NR3* sequence (sequence ID: CAMPEP_0187150166 in combined assembly peptides) obtained from the Marine Microbial Eukaryote Transcriptome Sequencing Project^29^ (MMETSP), except that the sequence obtained from cloned cDNA included 7 sites that were undetermined in the original sequence, 1 additional amino acid at site 166, and 15 additional amino acids (HASSQEVFNNESTTA) within the hinge 1 region at site number 418 to 432 (Supplementary Data S1). A region of the sequence spanning from the DI domain to the hinge 2 region showed that the deduced amino acid sequence of *NR3* lacked both the 2/2 HbN domain present in NR2 as well as the cytb-5 (Heme-Fe) binding domain found in other NRs (Fig. 2).

### Phylogenetic analysis

**Figure 3:**
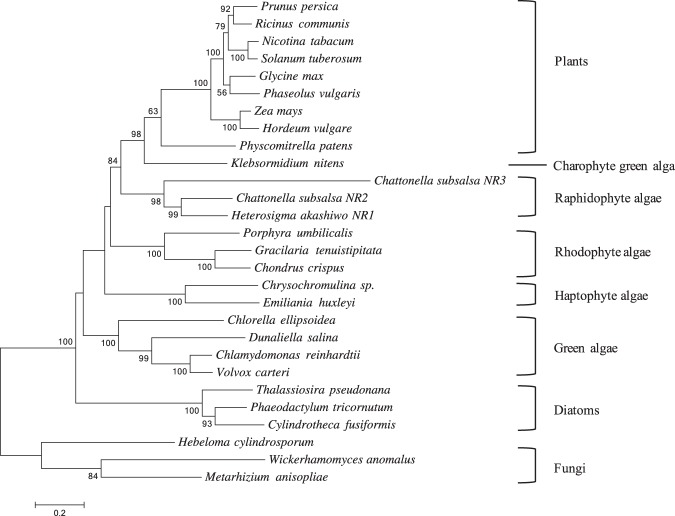
Molecular phylogenetic analysis of NR amino acid sequences in plants and algae. The tree was generated using the Maximum Likelihood method based on the Le Gascuel 2008 model^76^. The percentage of trees in which the associated taxa clustered together is shown next to the branches. Initial tree(s) for the heuristic search were obtained automatically by applying Neighbor-Join and BioNJ algorithms to a matrix of pairwise distances estimated using a JTT model, and then selecting the topology with superior log likelihood value. A discrete Gamma distribution was used to model evolutionary rate differences among sites (+G) and allowed for some sites to be evolutionarily invariable (+I). The tree is drawn to scale, with branch lengths measured in the number of substitutions per site. All positions containing gaps and missing data were eliminated. There were a total of 568 positions in the final dataset. Evolutionary analyses were conducted in MEGA7^72^. The tree was rooted by fungal NRs from *Wickerhamomyces anomalus*, *Hebeloma cylindrosporum*, and *Metarhizium anisopliae*. Bootstrap values less than 50 are not shown.

Phylogenetic analysis of NR3 in *C*. *subsalsa* revealed a close relationship to *Cs*NR2 and *Hs*NR1, with high bootstrap support (Fig. 3). Note that *Hs*NR1 and *Hs*NR2 are identical except for insertion of the 2/2 hemoglobin domain in *Hs*NR2. *Emiliania huxleyi* NR was clustered with NR of its haptophyte relative *Chrysochromulina*, and *Porphyra umbilicalis* NR clustered with NR sequences of other rhodophyte species *Gracilaria tenuistipitata*, and *Chondrus crispus*, all with high bootstrap support. Interestingly, *Klebsormidium nitens* NR is more closely related to NR in terrestrial plants than with other algal species. Phylogenetic analysis suggests that *Heterosigma* and *Chattonella* NRs may be more similar to NRs in terrestrial plants, as noted for *Hs*NR1 analysis by Coyne^14^, than to NRs found in other algal species, although the bootstrap support was low.

Evolutionary divergence between the deduced amino acid sequence of NR3 and NRs of other species indicates that NR3 is 49.82% and 49.65% identical to *Hs*NR1 and *Cs*NR2, respectively. Similar to results indicated by phylogenetic analysis, NR3 shares higher identity with *Klebsormidium nitens* NR (46.83%) and plant NRs (e.g. 44.19% identical to *Glycine max* NR) than to other algal NRs. When compared to algal NRs, for instance, NR3 is 37.68% and 40.32% identical to NR in *Thalassiosira pseudonana* (diatom) and *Chlamydomonas reinhardtii* (green alga), respectively.

### Diel expression of NR genes under different light conditions

**Figure 4:**
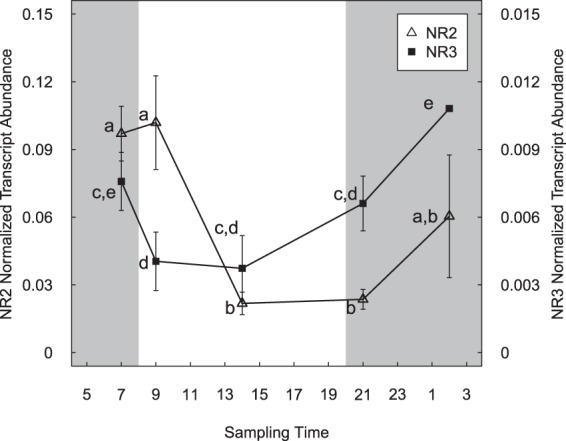
Transcript abundance of *NR2* (y axis shown at left) and *NR3* (y axis shown at right) normalized to actin in *C*. *subsalsa* cultured in a 12:12 h light: dark cycle. The shaded portion on the graph indicates the dark period. Different letters beside the symbols indicate significant differences in *NR* transcript abundance between each time point (p < 0.05). Error bars are standard deviation of three biological replicates.

Treatments were sampled at five time points from *C*. *subsalsa* cultures growing in a 12:12 h light to dark cycle (L:D, Fig. 4) or constant light (L:L). There were no significant changes in gene expression for either *NR2* or *NR3* over the five sampling time points for cultures in L:L treatments (p > 0.05, data not shown), although the lowest point measured for *NR2* gene expression coincided with that for the L:D treatment. *NR2* gene expression was significantly (3–30 fold) higher than *NR3* gene expression at all time points in each treatment (p < 0.05). Both *NR2* and *NR3* transcript levels exhibited diel expression patterns in the L:D treatment, but this pattern differed between *NR2* and *NR3*. There was a significant difference in *NR3* transcript abundance in samples collected in the light compared to the dark phase (p < 0.05), but no significant differences in *NR2* gene expression between light and dark phases (p > 0.05). *NR3* gene transcript abundance decreased significantly (p < 0.05) at one hour after lights on compared to the initial time point in the dark phase, and exhibited a trend (although not significant) toward increased expression of *NR3* after entering the dark phase. In addition, there was a significant increase (p < 0.05) in *NR3* transcript abundance at six hours after dark compared to six hours after lights on in the light phase. In contrast, there was no significant difference in *NR2* transcript abundance between the first two time points, with the change from dark to light phase (p > 0.05). There was a significant decrease in *NR2* transcript levels at six hours after lights on compared to 1 hour after lights on in the light phase (p < 0.05), and transcript abundance remained low at one hour after the start of the dark period before a trend to increasing expression at six hours after dark (not significant, p > 0.05).

### Gene expression of NRs with nitrate or ammonium as a nitrogen source

**Figure 5:**
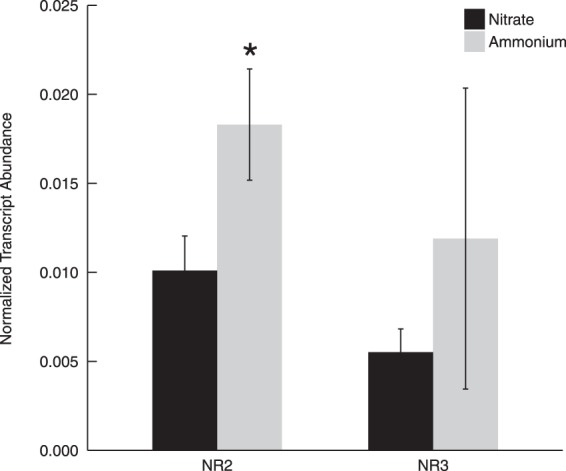
*NR2* and *NR3* transcript abundance normalized to actin in treatments cultured with nitrate or ammonium as the sole nitrogen source. Asterisk (*) reveals the significant difference between *NR* gene expression in nitrogen- treatments (p < 0.05). Error bars are standard deviation of three biological replicates.

Both *NR2* and *NR3* transcripts were detected in treatments with either NO_3_^−^ or NH_4_^+^ as the sole nitrogen source (Fig. 5). *NR2* had a significantly higher gene expression in treatments cultured with ammonium than with nitrate (p < 0.05), while gene expression of *NR3* was not significantly affected by nitrogen source (p > 0.05).

### Gene expression of NRs at different temperatures

**Figure 6:**
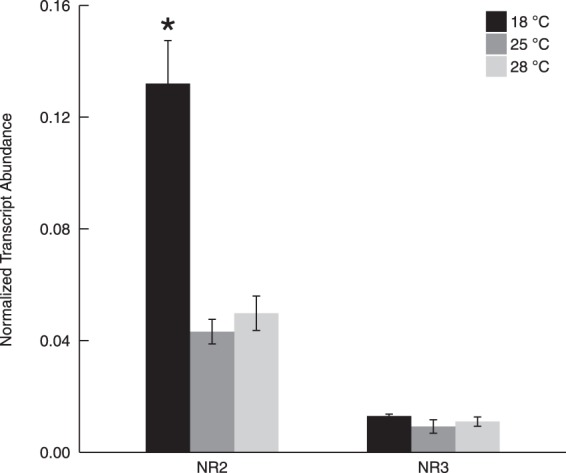
*NR2* and *NR3* transcript abundance normalized to actin at 18°, 25°, and 28 °C. Asterisk (*) indicates a significant difference in *NR* gene expression between treatments for each transcript (p < 0.05). Error bars represent standard deviation of three biological replicates.

*NR2* gene expression was significantly (3.0- and 2.6- fold) higher in the cultures acclimated to 18 °C than those in 25° and 28 °C, respectively (p < 0.05; Fig. 6). There was no significant difference in *NR2* gene expression for *C*. *subsalsa* cultured in 25° and 28 °C (p > 0.05). *NR3* gene expression was not significantly affected by temperature (p > 0.05). Furthermore, *NR2* gene expression values were significantly (4.5–10-fold) higher than *NR3* gene expression under all temperature conditions (p < 0.05).

### Identification of 14-3-3 binding motifs

Alignment of the hinge 1 region of NR3 revealed the presence of a serine- and proline-rich region consistent with 14-3-3 binding motifs in plant NRs. The binding motif for 14-3-3 proteins in NR3 was predicted by Scansite 3^30^ within the larger sequence SNRSRRL**S**APALPLA, with the potential phosphorylation site at the Ser446 (underlined in the sequence) in the hinge 1 region of *NR3* (Fig. 2). The search was rated in the top 0.147% of all sequence scores on Scansite 3. The predicted surface accessibility for this site within NR3 was 0.7828.

A total of 514 algal NR sequences in the NCBI database^31^ were downloaded and full-length algal NR sequences were selected for analysis (Supplementary Table S1). Among these 80 sequences, 12 were identified with at least one potential 14-3-3 binding motif as RXXSXP or KSXSXP. Full length algal NR sequences with putative 14-3-3 binding motifs were submitted for analysis using Scansite 3^30^. Five algal NRs, in addition to *C*. *subsalsa*, were identified by Scansite 3 to include canonical 14-3-3 binding motifs (Supplementary Table S2). Three of these were identified with high stringency: the charophyte green algal species, *Klebsormidium nitens* (GAQ91960.1), the haptophyte coccolithophore, *Emiliania huxleyi* (DAA12507.1), and the rhodophyte macroalgal species, *Porphyra umbilicalis* (OSX81470.1).

Two potential 14-3-3 binding motifs were identified in *Klebsormidium nitens* NR, within the sequences PTMKRSV**S**QPRMAAV and RGGSRFL**S**APTLDPL. These sequences were located in the hinge 1 and 2 regions with putative phosphorylation sites at S549 and S654 (underlined in the sequences above), respectively, and were rated in the top 0.094% and 0.068% of sequence scores, respectively. The putative 14-3-3 protein binding motif in the hinge 1 region had a surface accessibility of 1.60, while the motif in hinge 2 region had a low surface accessibility of 0.49 (Supplementary Table S2).

The 14-3-3 binding motif in NR of *Emiliania huxleyi* was identified within the sequence SAHLRAP**S**LPLGMRR with putative phosphorylation site at S639 (underlined) in the hinge 2 region. The search was rated in the top 0.068% on the site, and the surface accessibility was 0.50 (Supplementary Table S2).

The potential 14-3-3 binding motif identified in *Porphyra umbilicalis* included a phosphorylation site at S604 (underlined) in the hinge 1 region within the sequence ETLKRAK**S**APQINKM. The putative 14-3-3 protein binding site was rated in the top 0.015% on the site, with a high surface accessibility of 1.68 (Supplementary Table S2).

In addition to the three sequences above identified by a high stringency search, putative 14-3-3 protein binding motifs were also found in the hinge 1 region of NR sequences in rhodophytes *Gracilaria tenuistipitata* (ACX31653.1) and *Chondrus crispus* (XP_005714488.1) using a medium stringency setting in Scansite 3. A 14-3-3 binding site was predicted within the large sequence of KSG**S**APSLSTM at S523 in *G*. *tenuistipitata*, with a surface accessibility of 0.8526, and within the sequence of KSP**S**APALSS in *C*. *crispus* at site S526, with a surface accessibility of 1.0043 (Supplementary Table S2).

## Discussion

Blooms caused by the genus *Chattonella* have been responsible for massive fish kills in Japan, Korea, India, China, USA, and South Australia, with severe damage to fishery industries and economy^32^. Previous research identified temperature, salinity, irradiance, and nutrients as the most important factors to stimulate the growth and bloom formation of *Chattonella* spp.^32^. As both nitrate and ammonium contribute to growth of *Chattonella*^33^, the presence and relative concentrations of these nitrogen sources along with other environmental factors (such as temperature) that regulate NR likely play a key role in nitrogen assimilation and bloom formation by these species. In contrast to *Hs*NR1 and *Hs*NR2 in *Heterosigma akashiwo*, which are identical except for insertion of a 2/2HbN domain in *Hs*NR2^2,14^, analysis of NR amino acid sequences in *C*. *subsalsa* show that NR3 is distinct from *Cs*NR2 (Fig. 2) and suggests that these enzymes may be differentially regulated. A primary goal of this study was to investigate the transcriptional regulation of these two novel nitrate reductase enzymes in *C*. *subsalsa*.

Regulation of NR gene expression by light and/ or the circadian rhythm has been well established for other plants and algae but differ in a species-specific manner. For instance, NR gene expression was regulated by diel changes in light but not the circadian rhythm in diatom *Thalassiosira pseudonana*^34^, while it was regulated by the endogenous rhythm or both in plants *Arabidopsis thaliana*^35^, *Nicotiana tabacum* L^36^. and *Zea mays*^37^, as well as the red alga *Gracilaria tenuistipitata*^12^. Studies show that regulation of NR gene expression by light was related to negative and positive feedback loops, where the pool of amino acids are thought to play an important role in this process^16^. In addition to metabolic feedback, NR may be under circadian control via the central clock, which allows organisms to predict daily (and seasonal) changes in their environment and metabolic needs in order to optimize their growth^38^.

In this study, both *NR2* and *NR3* gene expression in *C*. *subsalsa* exhibited significant changes over time when cultured in a 12 h:12 h L:D cycle (Fig. 4). This pattern was abolished when the cultures were exposed to continuous light in the L:L treatment, indicating that gene expression for both *NR*s in this species were not constrained by an endogenous rhythm. Gene expression patterns between *NR2* and *NR3* in the L:D treatments, however, show that *NR3* gene expression was more responsive to changes in light, with a significant decrease in transcript abundance at one hour after lights on in the light phase compared to one hour before lights on in the dark phase. The slower response of *NR2* to the change from dark to light in the L:D treatment suggests a lower level of sensitivity to negative feedback loops regulating the expression of this gene. At six hours after the start of the light phase, both *NR2* and *NR3* transcript levels were at their lowest level. This decrease in transcript abundance during the light phase is similar to gene expression patterns for NR observed in other algal species^12,34^, and is consistent with studies demonstrating direct or indirect feedback repression of *NR* transcription by products of nitrogen assimilation such as amino acids^16^. Strong repression of *NR* gene expression by glutamine, for example, has been reported in plant species *Fagus sylvatica*^39^ and *Nicotiana plumbaginifolia*^40^, as well as in the red alga *Cyanidioschyzon merolae*^41^. The lack of a circadian rhythm in *C*. *subsalsa* is in contrast to *NR* gene expression patterns observed in the raphidophyte, *H*. *akashiwo*, where preliminary experiments indicate that *HsNR1* transcript abundance is under circadian control^14^. Overall, these results indicate multiple mechanisms regulating *NR* transcription between and among algal species.

*NR* gene expression in algal species typically increases with addition of nitrate, and can be strongly repressed by the presence of ammonium in some species^6^. NR gene expression was repressed or completely inhibited by ammonium in the green alga *Dunaliella tertiolecta*^26^, diatom *Cylindrotheca fusiformis*^42^, and red alga *Cyanidioschyzon merolae*^43^, for instance, although repression of *NR* gene expression by ammonium is not universal^7^. Zhang *et al*.^33^ found that *C*. *subsalsa* reached higher cell density in cultures supplied with ammonium as a nitrogen source compared to nitrate, but it was not clear if NR is repressed in this species by the presence of ammonium. In this study, *NR2* and *NR3* transcripts in *C*. *subsalsa* were detected in all treatments, even when ammonium was the sole nitrogen source (Fig. 5). The lack of repression by ammonium was also observed for *NR1* transcription in *H*. *akashiwo*^14^. Interestingly, the transcript abundance of *NR2* in *C*. *subsalsa* was significantly higher when growing with ammonium than with nitrate in this study, while *NR3* gene expression was not significantly different with either nitrogen source. The increased transcript abundance for *NR2* in ammonium cultures may reflect differences in cellular nutrient status associated with growth stage for these cultures compared to those provided with nitrate as a nitrogen source. Differential regulation of other genes involved in nitrogen metabolism by internal nitrogen status vs. external nitrogen source and concentrations has been observed in other algal species. The chloroplastic form of glutamine synthetase (GSII), for example, is stimulated by the presence of nitrate in the medium, while the mitochondrial form (GSIII) is expressed in response to ammonium derived from cellular processes^7,44^.

*C*. *subsalsa* is typically observed in Delaware’s inland bays at temperatures between 16 °C and 30 °C^33^. In this study, NR transcript abundance was measured after acclimation to temperatures that this species would experience in the field, and revealed a higher level of *NR2* gene expression at 18 °C compared to 25 °C and 28 °C, while *NR3* transcript abundance remained unchanged (Fig. 6). Parker and Armbrust^4^ suggested that photosynthesis and Calvin cycle efficiency in diatoms decrease at lower temperatures, and that increased rates of nitrate reduction under this condition may serve to dissipate excess energy in high light. Lomas and Glibert^45^ also suggested that diatoms took up nitrate in excess of nutritional requirements at lower temperatures, and used nitrate reduction as an electron sink when the harvesting and utilization of light energy were imbalanced. While we did not measure growth rates in this study, previous work by Zhang *et al*.^33^ found that *C*. *subsalsa* had a significantly reduced growth rate at 16 °C compared to 20 °C. The elevated *NR2* transcript abundance at lower temperatures measured here, along with reduced growth rates reported by Zhang *et al*., suggests that nitrate reduction may act as an energy sink in *C*. *subsalsa* when activities of other enzymes are not sufficient to sustain growth at lower temperatures. This is in contrast to the gene expression of *HsNR1* in *H*. *akashiwo*^14^, which was not significantly affected by the temperatures ranging from 18 ° to 28 °C, and maintained growth rates at 16 °C^33^, suggesting that nitrogen acquisition strategies may differ for these co-occurring species over this temperature range.

Proteins in the 14-3-3 family regulate widespread cellular processes in eukaryotes, such as intracellular signaling and control of cell cycles^46–49^. Under adverse conditions (e.g. low light), 14-3-3 proteins bind to the phosphorylated serine residue of plant NRs and block the electron flow from its NADH domain at the C-terminus to the Mo-MPT domain at the N-terminus, thus inhibiting NR activity and preventing the accumulation of toxic nitrite^50^. The 14-3-3 binding motif is highly conserved in plant NRs^6,20^. 14-3-3 proteins bind to a phosphorylated Ser residue within the motifs RXXpSXP as originally described by Muslin *et al*.^51^, or KSXpSXP in angiosperms^52^, where X in the sequence indicates any amino acid, and pS is the phospho-Ser residue^53^. Although the 14-3-3 binding motifs have not been reported in algal NR, evidence of indirect regulation of NR activity by 14-3-3 binding has been reported. In the green alga *Chlamydomonas*, for example, nitrogen assimilation is regulated by interaction of 14-3-3 proteins with glutamine synthetase I, indirectly affecting NR activity by feedback inhibition^23,54^.

Here, a putative 14-3-3 binding motif was found in the deduced amino acid sequence of NR3 in *C*. *subsalsa*. Regulation of NR3 by 14-3-3 binding proteins was also supported by the presence of a transcript encoding a 14-3-3 protein in the *C*. *subsalsa* transcriptome (ID: CAMPEP_0187155116). Putative 14-3-3 binding motifs were also identified in NRs of other algal species, *Klebsormidium nitens*, *Emiliania huxleyi*, and *Porphyra umbilicalis*, with a high stringency, and in NRs of *Gracilaria tenuistipitata* and *Chondrus crispus* with a medium stringency. Interestingly, 14-3-3 binding motifs identified in *C*. *subsalsa* and other algal species are all located in the hinge regions of these algae, consistent with 14-3-3 binding motifs in plant NRs. 14-3-3 binding motifs located within the hinge 1 region of *C*. *subsalsa*, *K*. *nitens*, *P*. *umbilicalis*, *G*. *tenuistipitata*, and *C*. *crispus* in particular, had surface accessibility scores indicating a higher probability of interactions with 14-3-3 binding proteins (Supplementary Table S1).

NRs with 14-3-3 binding motifs in the three rhodophyte species *P*. *umbilicalis*, *G*. *tenuistipitata*, and *C*. *crispus* are phylogenetically divergent from NRs in raphidophyte *C*. *subsalsa*, charophyte *K*. *nitens*, and haptophyte *E*. *huxleyi* (Fig. 3). If these NRs are regulated by 14-3-3 binding as in plants, this mode of regulation may have evolved independently among these algal species, or lost among other lineages over time. Interestingly, NR in *K*. *nitens* is closely related to NRs of terrestrial plants, consistent with a previous study showing that the *K*. *nitens* genome has a number of genes that were shared with land plants^55^. Although there have been no reported studies of NR regulatory mechanisms in *P*. *umbilicalis* and *C*. *crispus*, regulation of NR transcription in *G*. *tenuistipitata*^12^ and NR enzyme activity in both *G*. *tenuistipitata*^56^ and in *Gracilaria chilensis*^57,58^ have been investigated. NR activity in *G*. *tenuistipitata* was regulated by light and its circadian clock^56^. Intriguingly, NR in *G*. *chilensis* was regulated at the post-translational level by nitrogen source and light, involving phosphorylation and dephosphorylation^57,58^, providing for the possibility of NR regulation by 14-3-3 binding in this species. The amino acid sequence of NR in *E*. *huxleyi* and its gene expression responsive to nitrogen source were described by Bruhn *et al*.^59^. This enzyme was also characterized by Iwamoto and Shiraiwa^60^, showing that *E*. *huxleyi* NR is a hexamer and is NADH-specific. However, the post-translational regulation of *E*. *huxleyi* NR activity is not clear. Future investigations involving post-translational regulation of NRs in these species and the potential role of 14-3-3 binding motifs are required.

In addition to the presence of a putative 14-3-3 binding motif in *C*. *subsalsa* NR3, the deduced amino acid sequence lacks the conserved Heme-Fe domain (cytochrome b5) found in all other NRs (Fig. 2). This domain is considered one of the three strictly conserved functional domains (Heme-Fe, FAD, and Molybdenum cofactor) in eukaryotic NRs^6,61^. These enzymes are members of sulfite oxidase (SO) family^62,63^, which differs in structure between plants and animals. Similar to eukaryotic NRs, SO in animals contains a Heme-Fe domain^64^. The deduced amino acid sequence of NR3 in *C*. *subsalsa*, however, is more similar to plant SO which lacks the Heme-Fe domain^64^, preventing intramolecular electron transfers between redox centers. Instead of utilizing Fe(III) and cytochrome c as electron acceptors as occurs in animal SOs, plant SO uses molecular oxygen as the terminal electron acceptor^63,65^. It has also been shown that eukaryotic NRs have partial activities with fragments of different domains, using alternative electron donors and acceptors^15,66^, lending support for the function of NR3 in *C*. *subsalsa* without requiring the Heme-Fe domain.

The structure of NR may also make it possible for NR3 to function without the Heme-Fe domain. NRs are homodimers where dimerization is necessary for function, and further formation of a tetramer is a tendency by NR homodimers^67^. NR3 may participate in tetramer formation with *Cs*NR2, which includes both the Heme-Fe domain and a novel 2/2 hemoglobin domain. The additional heme-binding domain in *Cs*NR2 may complement the loss of the Heme-Fe domain in NR3 after formation of the multimeric protein. In addition to nitrate reduction and energy dissipation, NRs have also been reported to have alternative functions^5^, including participating in nitric oxide metabolism^2^, signaling^68^, and ATP generation^69^. Fully elucidating the function of NR3 will require additional study.

In summary, results of this study demonstrate a range of transcriptional responses to diel light cycles, nitrogen source, and temperature that act to differentially regulate *NR* gene expression in *C*. *subsalsa*. Transcription of *NR2* and *NR3* were differentially regulated by diel changes in light intensity, while *NR2* gene expression was also responsive to nitrogen source and temperature. Sequence analysis of the deduced amino acid sequence of *Cs*NR3 revealed the existence of a putative 14-3-3 binding motif in the hinge 1 region, suggesting the potential for post-translational regulation of this enzyme, as well as the lack of the Heme-Fe (cytochrome b5) domain. Comparisons between NR3 and plant SO suggest that alternative electron acceptors, or heterotetramer formation with *Cs*NR2, may permit this enzyme to function in the absence of Heme-Fe domain. To date, this is the first report of the presence of a putative 14-3-3 binding motif in algal NRs. A survey of NR sequences in other algal species, however, suggests that the presence of a putative 14-3-3 binding motif in *Cs*NR3 is not unique and that post-translational mechanisms regulating NR activity in plants may be shared with some algal species. Future research efforts may be directed to identify these mechanisms for a better understanding of environmental factors affecting nitrogen assimilation in algae.

## Materials and Methods

### Stock culture conditions

Stock cultures of *C*. *subsalsa* (National Center for Marine Algae and Microbiota, CCMP2191) were maintained in f/2 medium^70^ -Si at a salinity of 20 with either f/2 levels of nitrate (882 µM) or 100 µM ammonium. Cultures were incubated at 25 °C and 100 µmol m^−2^s^−1^ irradiance with cool fluorescent lights, and on a 12:12 h light: dark cycle.

### Identification and confirmation of NR3 sequence in *C*. *subsalsa*

The transcriptome of *C*. *subsalsa* (CCMP2191) was sequenced as part of the Marine Microbial Eukaryote Transcriptome Sequencing Project^29^ (MMETSP; Sample Name: MMETSP0947–0950; PI: Kathryn Coyne). A unique NR (designated NR3; ID: CAMPEP_0187150166; https://www.imicrobe.us/#/combined_assemblies/21) was identified and evaluated by BLASTX search^71^.

Plasmids designated CsNR3-P2 and CsNR3-P10 were prepared for sequencing to confirm NR3 sequence data obtained from MMETSP. Primers were designed to obtain overlapping segments of the entire NR3 cDNA sequence. RNA was extracted from stock cultures, and cDNA was prepared as described below. cDNA was diluted 1:20 with LoTE [3 mM Tris-HCl (pH 7.5), 0.2 mM EDTA] and used as template in PCR reactions. The PCR reactions included 1.0 µL diluted template, 0.2 mM dNTPs, 2.5 mM MgCl_2_, 1X Taq polymerase buffer (Sigma Chem. Co., St. Louis, MO, US), 0.25 units Jump-Start Taq Polymerase (Sigma Chem. Co.), and 0.25 µM of primers CsNR3 P2F and CsNR3 1228 R or CsNR3 1143 F and CsNR3 P10R. The PCR cycle consisted of 37 cycles of 30 s at 94 °C, 30 s at 58 °C, and 1.5 min at 72 °C. PCR products were cloned into pCR4 TOPO plasmid vector (Thermo Fisher Scientific Inc., Waltham, MA, US). Plasmids were prepared using QuickClean 5 M Miniprep Kit (GenScript Corporation, Piscataway, NJ, US) from liquid media following the manufacturer’s protocol.

Plasmids CsNR3-P2 and CsNR3-P10 were sequenced at Delaware Biotechnology Institute (University of Delaware, Newark, DE, US). The overlapping sequences were aligned with NR3 sequence from MMETSP using MEGA7^72^ (Supplementary Data S1). A consensus sequence of NR3 was deposited in GenBank (accession number: MH708883).

### Phylogenetic analysis

The deduced NR3 sequence was aligned with NR sequences of *Klebsormidium nitens* (GAQ91960.1), *Emiliania huxleyi* (DAA12507.1), *Porphyra umbilicalis* (OSX81470.1), *Gracilaria tenuistipitata* (ACX31653.1) and *Chondrus crispus* (XP_005714488.1), as well as other plant, fungal and algal NR sequences, including *Cs*NR2 (AER70127.1), *Hs*NR1 (ACS44801.1), NRs from plants [*Prunus persica* (BAB55002), *Ricinus communis* (AAG30576), *Nicotina tabacum* (CAA32216), *Solanum tuberosum* (BAB93534), *Glycine max* (AAA96813), *Phaseolus vulgaris* (P39866), *Zea mays* (AAD38068), and *Hordeum vulgare* (CAA40976)], moss [*Physcomitrella patens* (BAE19755)], haptophyte [*Chrysochromulina* sp. (KOO21257.1)], diatoms [*Phaeodactylum tricornutum* (AAV66996), *Cylindrotheca fusiformis* (AAY59538), and *Thalassiosira pseudonana* (EED88244.1)], green algae [*Chlorella ellipsoidea* (AAP32278), *Dunaliella salina* (AAP75705), *Chlamydomonas reinhardtii* (AAF17595), and *Volvox carteri* (AAA1114)], and fungi [*Wickerhamomyces anomalus* (AAF28059.1), *Hebeloma cylindrosporum* (CAB60010), and *Metarhizium anisopliae* (CAA04554)].

The sequences were aligned using Clustal W^73^ in Mega 7^72^, and the evolutionary divergence between these sequences were computed using p- distance method with complete deletion of gaps. The sequences included Mo-MPT, DI, FAD, and NAD(P)H domains, as well as hinge 1 and N-terminal regions. To build a phylogenetic tree, the best model was computed and selected in Mega 7^72^, then a phylogenetic tree was constructed using the LG + G + I model with a discrete Gamma distribution and 5 rate categories. The Maximum Likelihood method was used, and the tree was rooted by fungal NR sequences. Five hundred replicates were used to generate the bootstrap values.

### Diel expression of *NR* genes under different light conditions

*C*. *subsalsa* was cultured as above and cells were counted under a light microscope with a Sedgewick-Rafter chamber and inoculated into six replicate cultures at 11,400 cells mL^−1^. Three replicate cultures were moved into constant light at 100 µmol photons m^−2^ s^−1^ for 24 hours before the initial sampling and remained under constant light for the entire sampling period of 19 hours. The other replicate cultures (N = 3) were maintained on a 12:12 h light:dark cycle. Samples were collected at five time points: 1 hour before lights on (7:00), 1 hour after lights on (9:00), 6 hours after lights on (14:00), 1 hour after dark (21:00), and 6 hours after dark (2:00). At each time point, cultures were gently filtered onto 3 µm pore size polycarbonate filters (EMD Millipore Corporation, Billerica, MA, USA) for RNA extraction, described below.

### Gene expression of *NR*s with nitrate or ammonium as a nitrogen source

*C*. *subsalsa* was cultured as described under “Stock culture conditions” in f/2 medium with either 100 µM NO_3_^−^ or 100 µM NH_4_^+^ (N = 3). Semi-continuous cultures were maintained in exponential phase for several weeks (diluted every 3 to four days), and harvested during exponential phase growth at 6 hours after the start of the light cycle. Samples were collected as described above for RNA extraction. RNA was extracted as described below.

### Gene expression of *NR*s at different temperatures

*C*. *subsalsa* was cultured as described under “Stock culture conditions” at 18°, 25°, and 28 °C. Semi-continuous cultures (N = 3) were maintained in exponential phase for several weeks as described above before harvesting at 6 hours after the start of the light cycle. The samples were collected as above for RNA extraction. RNA was extracted as described below.

### RNA extraction and cDNA synthesis

RNA was extracted from filtered cells using the RNeasy Mini Kit (Qiagen, Chatswort, CA, US). Contaminating DNA was digested in 10 µl reaction volumes containing 300 to 1000 ng RNA and 1 µl DNase (100 units, Amplification Grade, Thermo Fisher Scientific Inc.) in 1X DNase I Reaction Buffer (Thermo Fisher Scientific Inc.) for 15 min at room temperature. The reaction was stopped by addition of EDTA (pH 8.0, Thermo Fisher Scientific Inc.), and the mixture was incubated at 65 °C for 15 min to degrade the DNase. The purified RNA was stored at −80 °C until use.

First strand cDNA was synthesized using the Superscript III First Strand Supermix Kit (Thermo Fisher Scientific Inc.) according to manufacturer’s directions using random hexamers to prime the reaction. The reaction was stopped by incubation at 85 °C for 5 min. The cDNA was stored at −80 °C until PCR analysis.

### Plasmid preparation

Plasmids were prepared from amplified cDNA to use as standards in qPCR analysis of NR2 and NR3. Diluted cDNA was used as template in PCR reactions with primers targeting *C*. *subsalsa NR2* and *NR3* (Table 1). PCR reactions were prepared as above with 0.075 µM CsNR3 1143 F and CsNR3 1228 R primers for CsNR3 amplification, or CsNR Glob 1005 F and CsNR Glob 1240 R for CsNR2 amplification. The PCR cycle consisted of 37 cycles of 30 s at 94 °C, 30 s at 53 °C, and 1 min at 72 °C.

**Table 1:** Sequence of *C*. *subsalsa*- specific primers for nitrate reductase and actin. F, forward primer; R, reverse primer.

Primers	Sequence (5′-3′)	Gene Targeted
CsNR Glob 1005 F	GAAGCTGCAATCCAAGCTGTAGT	NR2
CsNR Glob 1240 R	CCAACTCAGTCAACGTGTTCA	NR2
CsNR3 1143 F	CTGGACACAGCTGAGTTACA	NR3
CsNR3 1228 R	GCAATGCGATGAGTTGGAG	NR3
CsNR3 P2F	ATGGACGCATTCACATCCAGG	NR3
CsNR3 P10R	GAAAGTGAAGCACATTTCACGC	NR3
Cs Actin163F	GTGGGAGATGAGGCACAGTC	Actin
Cs Actin 294 R	TGCCACTCGAAGCTCATTGT	Actin

### qPCR analysis of *NR* gene expression

*NR*2 and *NR*3 gene expression was measured relative to actin transcript levels using quantitative real-time PCR (qPCR) on an ABI Prism 7500 Sequence Detection System (Thermo Fisher Scientific Inc.). Triplicate reactions included 5 µl Power SYBR^®^ Green PCR Master Mix (Thermo Fisher Scientific Inc.), 0.3 µM forward primer (CsNR Glob 1005 F for NR2, and CsNR3 1143 F for NR3), 0.3 µM reverse primer (CsNR Glob 1240 R for NR2, and CsNR3 1228 R for NR3), and 1 µl diluted (1:20) template cDNA. Primer sequences are in Table 1. Ten-fold dilutions of plasmids for CsNR2 and CsNR3 were used to make a standard curve of known copy numbers. Negative controls (without template) were run simultaneously. The cycling parameters were 50 °C for 2 min, and 95 °C for 10 min, followed by 40 cycles of 95 °C for 15 s, 56 °C for 30 s, and 60 °C for 1 min. The specificity of each reaction was evaluated by measuring the change in fluorescence with dissociation of duplex PCR products during a stepwise increase in temperature from 60 °C to 95 °C. The transcript copy numbers of *NR*2 and *NR*3 for each sample were determined by linear regression analysis, and then normalized to gene expression of actin (CAMPEP_ 0187146040) as an endogenous reference to produce a relative expression value for each *NR* in that sample.

### Statistical analysis

Repeated measures ANOVA was used to test if there were significant differences in gene expression over the entire sampling period. If the effect was significant (p < 0.05), then another repeated measures ANOVA was used to test the significant difference in transcript abundance in light vs. in dark (in L:D treatments), and paired t-test was conducted to analyze the significance of the differences between the values at each time point. Additionally, paired t-test was used to analyze the difference between *NR*2 and *NR*3 gene expression at each time point.

One-way ANOVA was used to test if nitrogen source and temperature had a significant effect on *NR* gene expression. If the effect was significant, Tukey’s HSD test was used to analyze the difference in all possible pairs. All statistical analyses were performed in R^74^ (V.3.2.4 Revised).

### Identification of 14-3-3 binding motifs

The translated amino acid sequence of NR3 was submitted to Scansite 3^30^ (http://scansite3.mit.edu; accessed on 2/28/2018) to identify potential 14-3-3 binding motifs in this sequence. Scansite is a computational tool to predict interactions between proteins and sites of phosphorylation. The parameters used in this search are listed here: group: phosphoserine/threonine binding; motif: 14-3-3 mode 1; and stringency: high.

NR sequences from other algal species were identified in the NCBI^31^ database (accessed on 11 Sept, 2017) and downloaded for analysis. Potential 14-3-3 binding sites including the motifs RXXSXP and KSXSXP were searched in 80 full-length algal NR sequences using MATLAB^75^ (V. R2016a). Sequences with putative 14-3-3 binding motifs were submitted to Scansite 3^30^ (http://scansite3.mit.edu) for further analysis, using parameters described above for *Cs*NR3 with high and medium stringency.

## Electronic supplementary material

Supplementary Tables

## Data Availability

All data generated during this study are available upon request.
