# Supplementary material for: Expression of novel nitrate reductase genes in the harmful alga, Chattonella subsalsa
Source: Sci Rep. 2018 Sep 7;8:13417. doi: 10.1038/s41598-018-31735-5 (PMC6128913; doi:10.1038/s41598-018-31735-5)
Supplement: Supplementary file 1 — Supplementary Tables [file 41598_2018_31735_MOESM1_ESM.pdf]

Expression of novel nitrate reductase genes in the harmful alga, *Chattonella subsalsa*

Yanfei Wang[1], Josée N. Bouchard[2], and Kathryn J. Coyne[1]*

[1]University of Delaware, 1044 College Drive, Lewes, DE 19958

[2] Algenol Biotech, 16121 Lee Rd., Suite 100, Ft. Myers, FL 33912

Email: kcoyne@udel.edu

Phone: 302-645-4236

Fax: 302-645-4007

ORCID: 0000-0001-8846-531X

Supplementary Data S1. Amino acid sequences of plasmids CsNR3-P2 (P2) and -P10 (P10) were aligned with NR3 sequence from Marine Microbial Eukaryote Transcriptome Sequencing Project (MMETSP). The overlapping parts in these sequences are indicated by "*", and the amino acids present in P2 or P10 sequence but absent in NR3 sequence from MMETSP is underlined.

```
C. subsalsa NR3 MMETSP MDAFTSRDSMKFEEMPSSNKNKLGFKEPKLENXIESGSITPISQHFCTSESLISDHDIDP
C. subsalsa NR3 P2     MDAFTSRDSMKFEEMPSSNKNKLGFKEPKLENLIESGSITPISQHFCTSESLISDHDIDP
C. subsalsa NR3 P10    ------------------------------------------------------------

C. subsalsa NR3 MMETSP SLLIDGAVERPNDLSVSSLTEMFQKCSFPVTLVDSGNRSNEVXGSKXHGSTWGPGAIGTS
C. subsalsa NR3 P2     SLLIDGAVERPNDLSVSSLTEMFQKCSFPVTLVDSGNRSNEVDGSKAHGSTWGPGAIGTS
C. subsalsa NR3 P10    ------------------------------------------------------------

C. subsalsa NR3 MMETSP IWSGIRLKDVLNHVGICDDDSNPKYIRFYSSEPDIISTSISVTVX-DPAADVLLAVEQNG
C. subsalsa NR3 P2     IWSGIRLKDVLNHVGICDDDSNPKYIRFYSSEPDIISTSISVTVVMDPAADVLLAVEQNG
C. subsalsa NR3 P10    ------------------------------------------------------------

C. subsalsa NR3 MMETSP EPLTPAHGGPLRVIVXGYVSGRSLKALRRIEVAAREADSPYHRTTNRFLPHQIDLEKALA
C. subsalsa NR3 P2     EPLTPAHGGPLRVIVPGYVSGRSLKALRRIEVAAREADSPYHRTTNRFLPHQIDLEKALA
C. subsalsa NR3 P10    ------------------------------------------------------------

C. subsalsa NR3 MMETSP DGWCDNPDTAINEFNXNSXISHPAHGQIISLDKMESALPVECRGYAYSGGGRKVVRIELS
C. subsalsa NR3 P2     DGWCDNPDTAINEFNVNSVISHPAHGQIISLDKMESALPVECRGYAYSGGGRKVVRIELS
C. subsalsa NR3 P10    ------------------------------------------------------------

C. subsalsa NR3 MMETSP VDDGQTWRPCSFSYEQGPTEAGKYWCWCLWSCQVPIMEFARSKQVMVRAFDSAWNTQPVS
C. subsalsa NR3 P2     VDDGQTWRPCSFSYEQGPTEAGKYWCWCLWSCQVPIMEFARSKQVMVRAFDSAWNTQPVS
C. subsalsa NR3 P10    ------------------------------------------------------------

C. subsalsa NR3 MMETSP PTPNLLGLMNNSIYSVKIYQDFLDTAELQFEHPGHKKTMGVETEAPTATISPTHRIA---
C. subsalsa NR3 P2     PTPNLLGLMNNSIYSVKIYQDFLDTAELQFEHPGHKKTMGVETEAPTATISPTHRIA---
C. subsalsa NR3 P10    ----------------------LDTAELQFEHPGHKKTMGVETEAPTATISPTHRIAHAS
                                             ***********************************

C. subsalsa NR3 MMETSP ------------ISAISRSNRSRRLSAPALPLASANPLTPPPLTKTFSSSSFSPPTLRKI
C. subsalsa NR3 P2     ------------------------------------------------------------
C. subsalsa NR3 P10    SQEVFNNESTTAISAISRSNRSRRLSAPALPLASANPLTPPPLTKTFSSSSFSPPTLRKI

C. subsalsa NR3 MMETSP GSKGNLRGRPHALNPKKKIPFRLIGKETLSYNTIRLRFALQSPNHVLGLPIGKHMFISAP
C. subsalsa NR3 P2     ------------------------------------------------------------
C. subsalsa NR3 P10    GSKGNLRGRPHALNPKKKIPFRLIGKETLSYNTIRLRFALQSPNHVLGLPIGKHMFISAP

C. subsalsa NR3 MMETSP IENRLCMRSYTPITGNEVDGYFDLIVKVYKPDPNFAEGGKMSQFLDQLSIGQSVDVKGPL
C. subsalsa NR3 P2     ------------------------------------------------------------
C. subsalsa NR3 P10    IENRLCMRSYTPITGNEVDGYFDLIVKVYKPDPNFAEGGKMSQFLDQLSIGQSVDVKGPL

C. subsalsa NR3 MMETSP GHIEYISNGNFVIHRKPFKVKNVLMLAGGTGITPMFQIMKAIMEDENDETNIYMIDANNS
C. subsalsa NR3 P2     ------------------------------------------------------------
C. subsalsa NR3 P10    GHIEYISNGNFVIHRKPFKVKNVLMLAGGTGITPMFQIMKAIMEDENDETNIYMIDANNS

C. subsalsa NR3 MMETSP EKDIILYNELQQLSEQNPRQCRIWHTILTPDDPETWCYDTGYVTAEMVRDHFPRPSSQSL
C. subsalsa NR3 P2     ------------------------------------------------------------
C. subsalsa NR3 P10    EKDIILYNELQQLSEQNPRQCRIWHTILTPDDPETWCYDTGYVTAEMVRDHFPRPSSQSL

C. subsalsa NR3 MMETSP VFMCGPSAMIHNACWPSLTKNGFKREMCFTF
C. subsalsa NR3 P2     -------------------------------
C. subsalsa NR3 P10    VFMCGPSAMIHNACWPSLTKNGFKREMCFTF
```

Supplementary Table S1. Full-length amino acid sequences of algal nitrate reductases (NRs) from NCBI database. The key words of “algae” and “nitrate reductase” yielded 514 sequences, including the 80 full length algal NRs below.

Accession Number	Species
AAA11144.1	*Volvox carteri*
AAC49459.1	*Chlorella vulgaris*
AAC49460.1	*Chlorella vulgaris*
AAF17595.1	*Chlamydomonas reinhardtii*
AAL79356.1	*Dunaliella tertiolecta*
AAP32278.1	*Pseudochlorella pringsheimii*
AAP75705.1	*Dunaliella salina*
AAT72293.1	*Dunaliella viridis*
AAT72294.1	*Dunaliella viridis*
AAV66996.1	*Phaeodactylum tricornutum*
AAY59538.1	*Cylindrotheca fusiformis*
ABJ91208.4	*Chlorella vulgaris*
ABO98391.1	*Ostreococcus lucimarinus* CCE9901
ABP97095.1	*Chlorella vulgaris*
ACF22999.1	*Chlorella vulgaris*
ACO68770.1	*Micromonas commoda*
ACS44801.1	*Heterosigma akashiwo*
ACX31652.1	*Gracilaria tenuistipitata*
ACX31653.1	*Gracilaria tenuistipitata*
AER70124.1	*Heterosigma akashiwo*
AER70125.1	*Heterosigma akashiwo*
AET85052.1	*Nannochloropsis sp.* W2J3B
AGC97428.1	*Dunaliella salina*
ASV49153.1	*Ulva prolifera*
ASV49154.1	*Ulva prolifera*
BAM79553.1	*Cyanidioschyzon merolae* strain 10D
CAA45497.1	*Volvox carteri f. nagariensis*
CBN78746.1	*Ectocarpus siliculosus*
CBX85127.1	*Chlorella vulgaris*
CBX85132.1	*Cylindrotheca fusiformis*
CBX85140.1	*Phaeodactylum tricornutum*
CBX85141.1	*Dunaliella viridis*
CBX85149.1	*Dunaliella salina*
CBX85151.1	*Pseudochlorella pringsheimii*

**Accession Number**	**Species**
CBX85159.1	*Dunaliella tertiolecta*
CBX85170.1	*Chlamydomonas reinhardtii*
CBX85213.1	*Volvox carteri*
CCO14816.1	*Bathycoccus prasinos*
CDF34669.1	*Chondrus crispus*
CDF34670.1	*Chondrus crispus*
DAA12507.1	*Emiliania huxleyi* CCMP1516
EDP00805.1	*Chlamydomonas reinhardtii*
EEC44781.1	*Phaeodactylum tricornutum* CCAP 1055/1
EED88244.1	*Thalassiosira pseudonana* CCMP1335
EEH58272.1	*Micromonas pusilla* CCMP1545
EFJ43675.1	*Volvox carteri f. nagariensis*
EFN52691.1	*Chlorella variabilis*
EIE21865.1	*Coccomyxa subellipsoidea* C-169
EJK46860.1	*Thalassiosira oceanica*
GAQ91960.1	*Klebsormidium nitens*
GAX09430.1	*Fistulifera solaris*
GAX24857.1	*Fistulifera solaris*
KIZ05991.1	*Monoraphidium neglectum*
KOO21257.1	*Chrysochromulina sp.* CCMP291
KXZ49738.1	*Gonium pectorale*
OEU19735.1	*Fragilariopsis cylindrus* CCMP1102
OLP85491.1	*Symbiodinium microadriaticum*
OLP86515.1	*Symbiodinium microadriaticum*
OLP86534.1	*Symbiodinium microadriaticum*
OLP89482.1	*Symbiodinium microadriaticum*
OLP99244.1	*Symbiodinium microadriaticum*
OLQ12636.1	*Symbiodinium microadriaticum*
OSX81470.1	*Porphyra umbilicalis*
OUS43413.1	*Ostreococcus tauri*
XP_001420098.1	*Ostreococcus lucimarinus* CCE9901
XP_001696697.1	*Chlamydomonas reinhardtii*
XP_002183599.1	*Phaeodactylum tricornutum* CCAP 1055/1
XP_002294410.1	*Thalassiosira pseudonana* CCMP1335
XP_002507512.1	*Micromonas commoda*
XP_002955156.1	*Volvox carteri f. nagariensis*
XP_003058321.1	*Micromonas pusilla* CCMP1545
XP_005535839.1	*Cyanidioschyzon merolae* strain 10D

Accession Number	Species
XP_005646409.1	*Coccomyxa subellipsoidea* C-169
XP_005714488.1	*Chondrus crispus*
XP_005714489.1	*Chondrus crispus*
XP_005844793.1	*Chlorella variabilis*
XP_007514576.1	*Bathycoccus prasinos*
XP_013905010.1	*Monoraphidium neglectum*
P36841.1	*Volvox carteri f. nagariensis*
XP_003081526.1	*Ostreococcus tauri*

Supplementary Table S2. Location and surface accessibility of potential 14-3-3 binding motifs in algal NRs predicted by Scansite 3 (Obenauer et al. 2003).

Species	Location	Surface accessibility
Chattonella subsalsa NR3	hinge 1 region	0.7828
Klebsormidium nitens	hinge 1 region	1.6
Klebsormidium nitens	hinge 2 region	0.49
Emiliania huxleyi	hinge 2 region	0.5
Porphyra umbilicalis	hinge 1 region	1.68
Gracilaria tenuistipitata	hinge 1 region	0.8526
Chondrus crispus	hinge 1 region	1.0043
